# A commentary on “Prognostic significance of the albumin-to-alkaline phosphatase ratio in gastrointestinal cancer patients: a systematic review and meta-analysis”

**DOI:** 10.1097/JS9.0000000000003284

**Published:** 2025-08-22

**Authors:** Jingyi Li, Bin Deng

**Affiliations:** Department of Gastroenterology, Northern Jiangsu People’s Hospital Affiliated to Yangzhou University, Yangzhou, Jiangsu, China

*Dear Editor,*

This article follows the TITAN Guideline^[1]^ and no artificial intelligence was used in the creation of the manuscript.

We read with interest the meta-analysis by Zhu et al^[2]^ addressing the prognostic role of the albumin-to-alkaline phosphatase ratio (AAPR) in gastrointestinal (GI) cancers. By synthesizing eight studies (*n* = 2267), the authors conclude that low AAPR predicts poor overall survival (OS)—a finding that positions AAPR as a cost-effective serological biomarker. This work aligns well with the oncology community’s need for accessible prognostic tools, and we commend the authors’ adherence to PRISMA/TITAN guidelines. That said, several limitations warrant careful consideration as they impact clinical translation.

Beyond the important findings, the retrospective design of all included studies introduces inherent biases that require acknowledgment. Selection bias and unmeasured confounders remain unavoidable in such analyses, particularly regarding factors influencing albumin levels—socioeconomic status, comorbidities, and healthcare access. Retrospective cohorts also risk overrepresenting patients with protocol-driven follow-up, potentially excluding those lost to early mortality like metastatic or frail subgroups. Consequently, AAPR’s prognostic value might be inflated if high-risk populations with hypoalbuminemia remain underrepresented. Future work should therefore prioritize prospective, population-based designs to better capture real-world heterogeneity^[3]^.

Compounding these design limitations, methodological heterogeneity in AAPR cutoff values presents significant challenges for clinical implementation. The substantial variation in thresholds (0.37–0.70) undermines clinical utility, likely stemming from non-standardized preanalytical conditions including fasting status and assay platforms, combined with arbitrary percentile-based dichotomization. As Altman et al^[4]^ noted, non-prespecified cutoffs can inflate biomarker performance by 30–60%. We consequently urge context-specific threshold derivation via ROC analysis in training cohorts, followed by independent validation to establish reproducible standards.

This methodological variability becomes particularly problematic when examining recurrence endpoints, where the analysis suffers from inadequate statistical power. The non-significant multivariate association between AAPR and recurrence-free survival (RFS; HR = 1.25, 95% CI: 0.33–4.77) likely reflects underpowering (*n* = 348, two studies)—a limitation compounded by the biological disconnect between RFS (dependent on localized factors like surgical margins) and AAPR’s reflection of systemic inflammation. Missing data on adjuvant therapies, critical recurrence confounders, further clouds interpretation. Larger studies integrating AAPR with tumor-specific biomarkers such as ctDNA are clearly needed to clarify recurrence prediction^[5]^.

Adding to these concerns, generalizability issues emerge from limited population diversity across studies. With all included research originating from China and Turkey, genetic, environmental, and healthcare disparities affecting AAPR remain unexplored. Endemic malnutrition in resource-limited regions could amplify AAPR’s prognostic weight, while ethnic variations in ALP isoenzymes may alter ratios substantially. As Riaz, Irbaz B et al^[6]^ argued, validation in ethnogeographically diverse cohorts is essential for globally applicable biomarkers.

Despite these limitations, Zhu et al. provide compelling evidence for AAPR as a pragmatic prognostic tool. Addressing these gaps will accelerate its adoption in precision oncology.

## Data Availability

Yes.
